# Morbidity After Mechanical Bowel Preparation and Oral Antibiotics Prior to Rectal Resection: The MOBILE2 Randomized Clinical Trial

**DOI:** 10.1001/jamasurg.2024.0184

**Published:** 2024-03-20

**Authors:** Laura Koskenvuo, Pipsa Lunkka, Pirita Varpe, Marja Hyöty, Reetta Satokari, Carola Haapamäki, Anna Lepistö, Ville Sallinen

**Affiliations:** 1 Department of Gastroenterological Surgery, Helsinki University Hospital and University of Helsinki, Helsinki, Finland; 2 Department of Digestive Surgery, Turku University Hospital and University of Turku, Turku, Finland; 3 Department of Gastroenterological Surgery, Tampere University Hospital, Tampere, Finland; 4 Human Microbiome Research Program, Faculty of Medicine, University of Helsinki, Helsinki, Finland; 5 Applied Tumor Genomics, Research Programs Unit, University of Helsinki, Helsinki, Finland

## Abstract

**Importance:**

Surgical site infections (SSIs)—especially anastomotic dehiscence—are major contributors to morbidity and mortality after rectal resection. The role of mechanical and oral antibiotics bowel preparation (MOABP) in preventing complications of rectal resection is currently disputed.

**Objective:**

To assess whether MOABP reduces overall complications and SSIs after elective rectal resection compared with mechanical bowel preparation (MBP) plus placebo.

**Design, Setting, and Participants:**

This multicenter, double-blind, placebo-controlled randomized clinical trial was conducted at 3 university hospitals in Finland between March 18, 2020, and October 10, 2022. Patients aged 18 years and older undergoing elective resection with primary anastomosis of a rectal tumor 15 cm or less from the anal verge on magnetic resonance imaging were eligible for inclusion. Outcomes were analyzed using a modified intention-to-treat principle, which included all patients who were randomly allocated to and underwent elective rectal resection with an anastomosis.

**Interventions:**

Patients were stratified according to tumor distance from the anal verge and neoadjuvant treatment given and randomized in a 1:1 ratio to receive MOABP with an oral regimen of neomycin and metronidazole (n = 277) or MBP plus matching placebo tablets (n = 288). All study medications were taken the day before surgery, and all patients received intravenous antibiotics approximately 30 minutes before surgery.

**Main Outcomes and Measures:**

The primary outcome was overall cumulative postoperative complications measured using the Comprehensive Complication Index. Key secondary outcomes were SSI and anastomotic dehiscence within 30 days after surgery.

**Results:**

In all, 565 patients were included in the analysis, with 288 in the MBP plus placebo group (median [IQR] age, 69 [62-74] years; 190 males [66.0%]) and 277 in the MOABP group (median [IQR] age, 70 [62-75] years; 158 males [57.0%]). Patients in the MOABP group experienced fewer overall postoperative complications (median [IQR] Comprehensive Complication Index, 0 [0-8.66] vs 8.66 [0-20.92]; Wilcoxon effect size, 0.146; *P* < .001), fewer SSIs (23 patients [8.3%] vs 48 patients [16.7%]; odds ratio, 0.45 [95% CI, 0.27-0.77]), and fewer anastomotic dehiscences (16 patients [5.8%] vs 39 patients [13.5%]; odds ratio, 0.39 [95% CI, 0.21-0.72]) compared with patients in the MBP plus placebo group.

**Conclusions and Relevance:**

Findings of this randomized clinical trial indicate that MOABP reduced overall postoperative complications as well as rates of SSIs and anastomotic dehiscences in patients undergoing elective rectal resection compared with MBP plus placebo. Based on these findings, MOABP should be considered as standard treatment in patients undergoing elective rectal resection.

**Trial Registration:**

ClinicalTrials.gov Identifier: NCT04281667

## Key Points

**Question:**

Does mechanical and oral antibiotic bowel preparation (MOABP) reduce morbidity in patients undergoing elective rectal resection?

**Findings:**

In this randomized clinical trial comparing MOABP with mechanical bowel preparation (MBP) plus placebo in 565 patients who underwent elective rectal resection with primary anastomosis, overall morbidity measured by the Comprehensive Complication Index was lower in the MOABP group. Rates of surgical site infection and anastomotic dehiscence were also lower in the MOABP group.

**Meaning:**

Findings of this study suggest that MOABP results in fewer postoperative complications than MBP alone and should be the standard regimen in patients undergoing elective rectal resection.

## Teaser

This randomized clinical trial compares the effectiveness of mechanical and oral antibiotics bowel preparation with that of mechanical bowel preparation alone for reducing morbidity in patients undergoing elective rectal resection.

## Introduction

Colorectal surgery carries a substantial risk of morbidity mainly caused by surgical site infections (SSIs), which can affect up to one-third of patients.^1,2^ Within the field of colorectal surgery, the risk of SSIs is higher in rectal surgery, especially if low colorectal or coloanal anastomoses are fashioned.^3^ In addition to short-term morbidity, postoperative complications may have long-term consequences that increase the risk of cancer metastases or local recurrence.^4^ While consensus and high-quality evidence regarding the benefits of prophylactic preoperative intravenous antibiotics exists,^5,6,7^ there is an ongoing debate as to whether preoperative oral antibiotics are beneficial in reducing the morbidity associated with rectal resection.

Several retrospective studies from the American College of Surgeons National Surgical Quality Improvement Program reignited the debate almost a decade ago and suggested that mechanical and oral antibiotic bowel preparation (MOABP) is associated with lower rates of SSIs in colorectal surgery compared with only mechanical bowel preparation (MBP) or no bowel preparation.^8,9,10,11,12^ A recent meta-analysis of randomized clinical trials (RCTs) found an approximately 50% reduction in SSIs when oral antibiotics are added to bowel preparation regimens.^13^ However, a crucial limitation of the RCTs included in the meta-analysis is that outcomes were not reported separately for rectal surgery. Rectal surgery is often preceded with neoadjuvant radiotherapy or chemoradiotherapy and, due to higher rates of anastomotic dehiscence especially in low anastomoses, a protective stoma is often used. While MBP is not considered necessary in colon surgery, in rectal surgery MBP diminishes SSIs and is widely prescribed.^14^ These limitations and the need of RCTs to focus on specific types and locations of colorectal resections, such as rectal resections, were also highlighted by a recent review.^15^ Furthermore, previous RCTs focused on SSIs only; however, it is important to evaluate overall cumulative complications, as oral antibiotics might have unintended adverse consequences.^16^ The global use of oral antibiotics prior to colorectal surgery is highly variable. About 10% of patients undergoing colorectal surgery in Germany receive oral antibiotics, while up to two-thirds of patients in the US are prescribed MOABP.^17,18^

To our knowledge, no high-quality, large, double-blind RCT assessing MOABP in rectal resection has been published. Because of variable practices and a lack of high-quality evidence regarding the use of oral antibiotics in rectal surgery, we designed and carried out the Mechanical Bowel Preparation and Oral Antibiotics vs Mechanical Bowel Preparation Only Prior to Rectal Surgery (MOBILE2) trial. We hypothesized that MOABP reduces overall complications after elective rectal resection with a colorectal or coloanal anastomosis compared with MBP alone.

## Methods

### Study Design

The MOBILE2 trial was a double-blind, placebo-controlled RCT conducted at 3 large university hospitals in Finland (Helsinki University Hospital, Turku University Hospital, and Tampere University Hospital) that serve over 70% of the Finnish population. The full trial protocol has been published^19^ and is presented in Supplement 1. There were no changes in the study protocol after the trial started, except for adding a subgroup analysis for surgical approach (minimally invasive or open surgery) based on peer reviewer comments on the protocol article.^19^ The change was made while the trial was still recruiting, and no data analyses had been performed. The research plan was evaluated by the Finnish National Committee on Medical Research Ethics and the Finnish Medicines Agency and was approved by the local ethics committee of Helsinki University Hospital and by each participating centers’ institutional review board. The trial was conducted in accordance with the principles of the Declaration of Helsinki^20^ and Good Clinical Practice. The trial was monitored by the Clinical Research Institute HUCH (HYKS-instituutti Oy; Helsinki, Finland) and followed the Consolidated Standards of Reporting (CONSORT) reporting guideline.

### Patient Eligibility

Patients scheduled for elective anterior rectal resection (including low or ultralow anterior rectal resection) with colorectal or coloanal anastomosis due to a rectal tumor between March 18, 2020, and October 10, 2022, were eligible for inclusion in the study. Tumors 15 cm or less from the anal verge on magnetic resonance imaging (MRI) (or on endoscopy if MRI was not performed) were considered rectal tumors. We used MRI as the primary method to measure distance from the anal verge. Additionally, MRI was the modality used for local tumor staging and computed tomography (CT) was used for assessing potential distant metastases. The exclusion criteria were: (1) emergency surgery, (2) bowel obstruction before the surgery, (3) ostomy created before rectal surgery, (4) any reason preventing MBP, (5) allergy to the antibiotics used in the study, (6) age younger than 18 years, or (7) inadequate ability to understand the study protocol and instructions. Postrandomization exclusion criteria were: (1) surgery was not performed, (2) rectal resection was not performed, or (3) colorectal or coloanal anastomosis was not created.

### Randomization and Masking

Patients were individually randomized in a 1:1 ratio to receive either MOABP or MBP plus placebo. The randomization sequence was generated via computer using variable block sizes (4, 6, and 8 patients). Oral antibiotics or placebo tablets were produced and packed into sealed vials at the Helsinki University Hospital pharmacy’s clinical trials unit. No person outside the pharmacy’s clinical trials unit had access to the randomization sequence. The external appearance of the placebo tablets was made identical to the oral antibiotics. The surgeons recruited patients. After the patients gave their written informed consent, they were given a numbered vial (containing either placebo or oral antibiotics) per the stratification group in numerical order. The patient population was stratified according to the distance of the lower edge of the tumor from the anal verge (measured from MRI scans of the rectum or at endoscopy) and the preoperative treatment they received. Four stratification groups were created: (1) tumor less than 10 cm from the anal verge and no preoperative treatment or short-course radiotherapy (SCRT) with immediate surgery; (2) tumor less than 10 cm from the anal verge and long-course chemoradiotherapy (LCCRT) or SCRT with a long waiting time or with chemotherapy before operation; (3) tumor 10 cm or more from the anal verge and no preoperative treatment or SCRT with immediate surgery; and (4) tumor 10 cm or more from the anal verge and LCCRT or SCRT with a long waiting time or with chemotherapy before operation.

Emergency envelopes with information regarding the allocation group were available in case this information was suddenly needed during treatment. These were not opened during the study and thus masking was maintained during the trial. All those involved in the study were blinded to the allocated treatment. After all of the data had been collected and verified, Helsinki University Hospital’s pharmacy clinical trials unit provided an unmasking list with the allocated groups designated with letters A and B. Only after the statistical analyses were performed were the final allocated groups revealed completely.

### Surgical Procedures

For MBP, all patients drank 2 L of polyethylene glycol and at least 1 L of any clear fluid. The MBP could be started 2 days before surgery at 3 PM and had to be completed by 3 PM on the day prior to surgery. After completing MBP, patients in the MOABP group were instructed to take 1 g of neomycin and 1 g of metronidazole orally at 3 PM and 11 PM on the day before surgery; patients in the MBP-only group took identical placebo tablets at these same times. Neomycin and metronidazole were chosen because of their broad spectrum of action and neomycin’s poor absorbability. Patient compliance was assessed by a preoperative nurse on the morning of the surgery by asking whether the patient had taken the pills at the specified times and recording this information in a case report form. All patients were to receive perioperative prophylactic intravenous antibiotics (cefuroxime, 1.5 g, and metronidazole, 500 mg; 3 patients received other antibiotics due to allergy [2 received ciprofloxacin and 1 received levofloxacin]) approximately 30 minutes before surgery. Diverting ostomy was used in patients with a low colorectal or coloanal anastomosis (<6 cm from anal verge) and was also allowed in patients with a higher anastomosis in case of a nonsuturable leak in the intraoperative air leak test, or if there were any reasons for a surgeon to consider a diverting ostomy necessary. At 6 to 8 weeks after surgery, patients were scheduled to undergo abdominal imaging with rectally administered contrast medium or a sigmoidoscopy, and the occurrence of any SSIs, other complications, reoperations, or death was assessed.

### Outcomes

The primary outcome was the Comprehensive Complication Index (CCI) within 30 days after surgery.^21^ The CCI is a continuous measure of cumulative postoperative complication burden and uses the Clavien-Dindo classification for individual complication classification. The CCI is scored from 0 (no complications) to 100 (death). As an example, a CCI of 8.7 points indicates 1 Clavien-Dindo grade I complication and a CCI of 33.7 points indicates 1 Clavien-Dindo grade IIIb complication. The secondary outcomes were: (1) SSI within 30 days after surgery, including superficial incisional, deep incisional, and organ or space infections (defined according to the Centers for Disease Control and Prevention criteria^22^) assessed on the ward by the surgeon responsible for the patient and recorded on a case report form and the patient’s medical record; during a reoperation; or, for organ or space infections, from a CT scan interpreted by an expert gastrointestinal radiologist; (2) the number and classification of anastomotic dehiscence (grade A: anastomotic dehiscence resulting in no change in patient management; grade B: anastomotic dehiscence requiring active therapeutic intervention but no repeat laparotomy; grade C: anastomotic dehiscence requiring repeat laparotomy or laparoscopy^23^) within 30 days after surgery; (3) length of hospital stay; (4) mortality within 90 days after surgery (any cause); and (5) the number of patients who received adjuvant treatment divided by the number of patients needing it. The criteria for adjuvant therapy were pathologic nodal positivity, lymphovascular invasion, high tumor budding, or high-grade adenocarcinoma. Anastomotic dehiscence of asymptomatic patients was defined as extravasation of contrast medium during CT as assessed by a radiologist or as a visible fistula cavity during flexible sigmoidoscopy as assessed by the colorectal surgeon. Potential adverse effects of antibiotics were recorded.

### Statistical Analysis

The sample size calculation was based on the baseline risk of deep incisional and organ or space SSIs in anterior rectal resections performed at Helsinki University Hospital between 2005 and 2011 (approximately 13%)^24^ and in our group’s previous trial that recruited patients for colon surgery (the MOBILE1 trial; approximately 6% to 8%).^25^ The mean (SD) CCIs in the 2 groups in the MOBILE1 trial were 9 (16) points and 10 (13) points. Therefore, we estimated that both the CCI and SD would be higher in patients undergoing rectal surgery than in those undergoing colon surgery. The sample size was calculated with the aim of showing a difference of 5 CCI points between the 2 groups (hypothesis: a mean [SD] 12.5 [18] points in the MOABP group and 17.5 [18] points in the MBP plus placebo group). With a power of 90% and a margin of error of 5%, 574 patients needed to be recruited (Wilcoxon-Mann-Whitney test). About 5% of patients were estimated to be excluded after randomization, resulting in a final sample size goal of 604 patients.^19^

Categorical variables were compared using the χ^2^ test, or Fisher exact test if fewer than 5 cases were expected in a cell. The effect size for categorical variables was estimated using odds ratios (ORs) with 95% CIs. Continuous variables with normal distribution were reported as means with SDs and were compared using the *t* test. Effect size for such variables was estimated by reporting the difference of means with 95% CIs. Continuous variables with nonnormal distribution were reported as medians with IQRs and compared using the Mann-Whitney *U* test; effect size was reported as the Wilcoxon effect size without 95% CIs. For transparent reporting and to ease future meta-analyses, means and SDs are also reported for the primary outcome. With Bonferroni correction, statistical significance was set at *P* < .01 for the secondary outcomes and a 2-sided α = .05 for the primary outcome. Statistical analyses were performed using SPSS Statistics, version 27.0 (IBM). Patients with missing data were excluded from analyses of that particular variable, and missing values were not imputed. The number of patients with missing values, if any, are stated within the tables or in the text when reporting the variable. Outcomes were analyzed using a modified intention-to-treat principle, which included all patients who were randomly allocated to and underwent elective rectal resection with an anastomosis. Subgroup analyses for primary (CCI) and first secondary outcomes (SSI) were prespecified and included (1) tumor location, (2) neoadjuvant treatment with LCCRT or SCRT with chemotherapy or a long waiting time before operation (yes or no), (3) protective ostomy (yes or no), and (4) surgical approach (minimally invasive or open surgery). With Bonferroni correction, statistical significance was set at *P* < .003125 for the subgroup analyses.

## Results

Between March 18, 2020, and October 10, 2022, 845 patients in the 3 participating centers were planned to have elective anterior rectal resection, and 765 patients were assessed for eligibility. In all, 610 patients were randomly allocated to either MBP plus placebo or MOABP (Figure). Trial recruitment stopped once the prespecified sample size was obtained. After exclusions, the modified intention-to-treat analyses included 565 patients, with 277 in the MOABP group (median [IQR] age, 69 [62-74] years; 190 males [66.0%] and 98 females [34.0%]) and 288 patients in the MBP plus placebo group (median [IQR] age, 70 [62-75] years; 158 males [57.0%] and 119 females [43.0%]). Patients’ baseline characteristics (Table 1), tumor characteristics (Table 2), neoadjuvant treatments (Table 2; eTable 3 in Supplement 2), and operative details (Table 3) were similar between the 2 groups. All patients received preoperative intravenous antibiotics and the timing of their administration before incision was similar between the groups (Table 3). One patient in the MOABP group and 2 patients in MBP plus placebo group did not undergo preoperative MRI.

**Figure.**
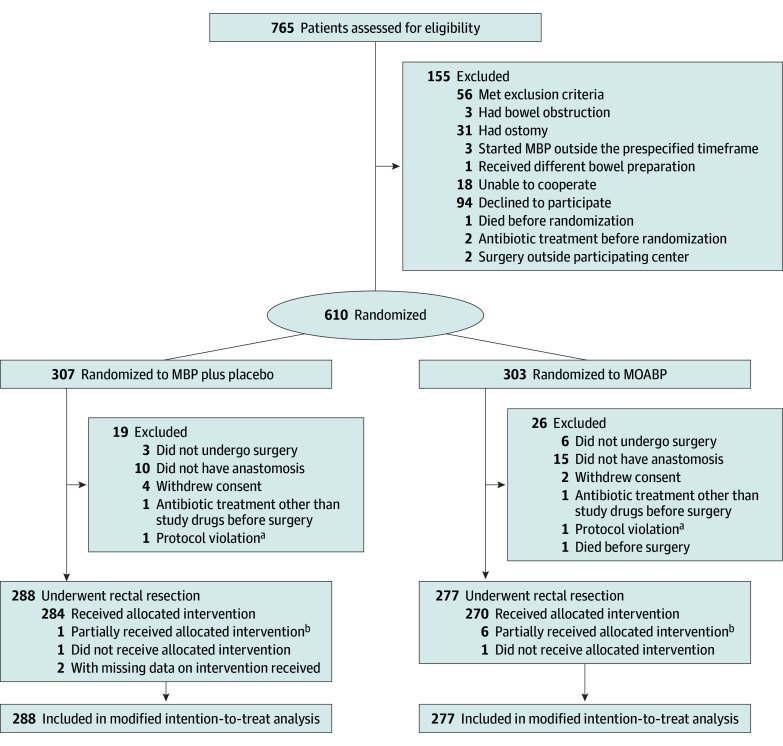
CONSORT Flow Diagram MBP indicates mechanical bowel preparation; MOABP, mechanical and oral antibiotics bowel preparation. ^a^The vial number in 1 patient in each group was not registered; hence, the medications that these patients received were unknown. ^b^Only some of the oral antibiotics or placebo tablets were taken by the patient, or only part of the MBP liquid was ingested.

**Table 1.** Baseline Characteristics of Patients in the MOABP and MBP Plus Placebo Groups

Characteristic	No. (%)^a^	
	MBP plus placebo (n = 288)	MOABP (n = 277)
Age, median (IQR), y	69 (62-74)	70 (62-75)
Sex		
Female	98 (34.0)	119 (43.0)
Male	190 (66.0)	158 (57.0)
BMI, median (IQR)	25.14 (22.99-28.40)	26.12 (23.27-29.18)
Hypoalbuminemia^b^	59 (21.6)^c^	60 (22.3)^d^
Anemia^e^	94 (32.6)	80 (28.9)
CEA, ng/mL	2.00 (1.2-3.5)^f^	2.20 (1.3-5.0)^g^
Smoker	44 (15.3)^h^	30 (10.9)
ASA physical status score^i^		
1	8 (2.8)	9 (3.2)
2	122 (42.4)	113 (40.8)
3	143 (49.7)	146 (51.6)
4	15 (5.2)	9 (3.2)
Comorbidities		
Coronary disease (not infarction)	20 (6.9)	26 (9.4)
Hypertension	93 (32.3)	114 (41.2)
Myocardial infarction	8 (2.8)	8 (2.9)
Congestive heart failure	10 (3.5)	6 (2.2)
Atrial fibrillation	28 (9.7)	27 (9.7)
ASO	6 (2.1)	5 (1.8)
CVA or TIA	14 (4.9)	12 (4.3)
Hemiplegia	1 (0.3)	0
Dementia	2 (0.7)	3 (1.1)
COPD or asthma	14 (4.9)	25 (9.0)
Connective tissue disease	7 (2.4)	4 (1.4)
Diabetes		
Without complications	35 (12.2)	45 (16.2)
With complications	3 (1.0)	3 (1.1)
Liver disease		
Mild	1 (0.3)	2 (0.7)
Moderate or severe	1 (0.3)	0
Kidney disease (moderate or severe)	4 (1.4)	6 (2.2)
Ventricular ulcer	0	1 (0.4)
Cancer	242 (84.0)	235 (84.8)
Metastatic malignancy	20 (6.9)	14 (5.1)
No comorbidities	20 (6.9)	21 (7.6)
Charlson Comorbidity Index^j^		
Mild (0-2)	198 (68.8)	187 (67.5)
Moderate (3-4)	62 (21.6)	68 (24.6)
Severe (≥5)	28 (9.8)	22 (7.9)
Score, median (IQR)	2.0 (2.0-3.0)	2.0 (2.0-3.0)
High-risk medication		
Aspirin	28 (9.7)	25 (9.0)
Clopidogrel	8 (2.8)	3 (1.1)
Warfarin	8 (2.8)	5 (1.8)
Low-molecular-weight heparin	4 (1.4)	4 (1.4)
Direct oral anticoagulant	14 (4.9)	19 (6.9)
≥2 Medications that affect thrombosis (antithrombotic or anticoagulant)	7 (2.4)	4 (1.4)
Immunosuppressive medication or corticosteroid	9 (3.1)	6 (2.2)
NSAID	4 (1.4)	1 (0.4)
No high-risk medication	206 (71.5)	210 (75.8)
Previous abdominal or inguinal operation	132 (45.8)	126 (45.5)

Abbreviations: ASA, American Society of Anesthesiologists; ASO, arteriosclerosis obliterans; BMI, body mass index (calculated as weight in kilograms divided by height in meters squared); CEA, carcinoembryonic antigen; COPD, chronic obstructive pulmonary disease; CVA, cerebrovascular accident; MBP, mechanical bowel preparation; MOABP, mechanical and oral antibiotics bowel preparation; NSAID, nonsteroidal anti-inflammatory drug; TIA, transient ischemic attack.

SI conversion factors: To convert CEA from nanogram per mililiter to microgram per liter, multiply by 1; to convert albumin to grams per liter, multiply by 10; to convert hemoglobin from grams per deciliter to grams per liter, multiply by 10.

^a^ Patients with missing data for each variable were not included in calculations.

^b^ Hypoalbuminemia defined as an albumin level less than 3.6 g/dL.

^c^ Data missing for 15 patients.

^d^ Data missing for 8 patients.

^e^ Anemia defined as a hemoglobin level less than 11.7 g/dL in females and less than 13.4 g/dL in males.

^f^ Data missing for 5 patients; only patients with rectal cancer (n = 261) included.

^g^ Data missing for 3 patients; only patients with rectal cancer (n = 250) included.

^h^ Data missing for 1 patient.

^i^ Scores range from 1 to 4, with 1 indicating a healthy patient and 4 indicating a patient with severe systemic disease that is a constant threat to life.

^j^ Scores range from 0 to 29, with higher scores indicating greater severity.

**Table 2.** Rectal Tumor and Neoadjuvant Therapy Characteristics of Patients in the MOABP and MBP Plus Placebo Groups

Characteristic	No. (%)^a^	
	MBP plus placebo (n = 288)	MOABP (n = 277)
Benign tumor	27 (9.4)	27 (9.7)
Rectal cancer TNM classification		
No. of patients^b^	261	250
T0/Tx	7 (2.7)	8 (3.2)
T1-T2	58 (22.4)	59 (23.7)
T3	131 (50.6)	114 (45.8)
T4	63 (24.3)^c^	68 (27.3)^d^
N0	114 (44.0)	97 (38.8)
N1	91 (35.1)	98 (38.2)
N2	54 (20.8)^e^	55 (22.0)
M0	248 (95.0)	238 (95.2)
M1	13 (5.0)	12 (4.8)
Rectal cancer stage		
No. of patients^b^	261	250
1	53 (20.5)	53 (21.2)
2	59 (22.8)	45 (18.0)
3	134 (51.7)	140 (56.0)
4	13 (5.0)^f^	12 (4.8)
Tumor height at endoscopy, median (IQR), cm	10.0 (7.0-12.0)^g^	10.0 (7.0-12.0)^h^
Tumor height at MRI, median (IQR), cm	8.35 (6.5-10.5)^i^	8.5 (6.5-10.0)^j^
Neoadjuvant treatment		
None	167 (64.0)	162 (65.7)
Short-course radiotherapy	39 (14.9)	32 (12.8)
Short-course radiotherapy with long waiting time	2 (0.8)	5 (2.0)
Long-course chemoradiotherapy	50 (19.2)	46 (18.4)
Short-course radiotherapy and chemotherapy	1 (0.4)	2 (0.8)
Chemotherapy only	2 (0.8)	3 (1.2)

Abbreviations: TNM, tumor, nodes, and metastases; MBP, mechanical bowel preparation; MOABP, mechanical and oral antibiotics bowel preparation; MRI, magnetic resonance imaging.

^a^ Patients with missing data for each variable were not included in calculations. Some TNM and tumor height in MRI data are missing because MRI was not taken, or tumor was not visible during MRI.

^b^ Based on preoperative computed tomography (M category) and MRI (T and N categories).

^c^ Data missing for 2 patients.

^d^ Data missing for 1 patient.

^e^ Data missing for 2 patients.

^f^ Data missing for 2 patients.

^g^ Data missing for 7 patients.

^h^ Data missing for 4 patients.

^i^ Data missing for 8 patients.

^j^ Data missing for 9 patients.

**Table 3.** Operative Details of Patients in the MOABP and MBP Plus Placebo Groups

Characteristic	No. (%)	
	MBP plus placebo (n = 288)	MOABP (n = 277)
Anterior resection and anastomosis type		
Colorectal anastomosis		
Partial TME	99 (34.4)	97 (35.0)
Total TME	165 (57.3)	160 (57.8)
Coloanal anastomosis	24 (8.3)	20 (7.2)
Surgical approach		
Open	217 (75.3)	210 (75.8)
Laparoscopic	28 (9.7)	26 (9.4)
Robotic	34 (11.8)	37 (13.4)
Laparoscopic or robotic converted to open	9 (2.8)	4 (1.5)
Perioperative details		
Timing of preoperative IV antibiotic administration before incision, median (IQR), min	26.0 (17.0-35.0)	27.0 (18.0-36.0)
Duration of operation, median (IQR), min	142.5 (119.0-186.0)	143.0 (119.0-180.0)
Estimated intraoperative blood loss, median (IQR), mL	150.0 (50.0-250.0)^a^	150.0 (50.0-200.0)
Anastomotic height from anal verge, median (IQR), cm	5.00 (4.0-7.0)	5.0 (4.0-7.0)
Protective loop ostomy		
Transversostomy	193 (67.0)	173 (62.5)
Ileostomy	29 (10.1)	23 (8.3)
No stoma	66 (22.9)	81 (29.2)

Abbreviations: IV, intravenous; MBP, mechanical bowel preparation; MOABP, mechanical and oral antibiotics bowel preparation; TME, total mesorectal excision.

^a^ Data missing for 2 patients; patients with missing data were not included in calculations.

Patients in the MOABP group had fewer overall postoperative complications (primary outcome) compared with patients in the MBP plus placebo group (median [IQR] CCI, 0 [0-8.66] vs 8.66 [0-20.92]; Wilcoxon effect size, 0.146; *P* < .001) (Table 4). A detailed list of all complications is shown in eTable 1 in Supplement 2. In total, 155 (56.0%) patients in the MOABP group and 134 patients (46.5%) in the MBP plus placebo group had no complications. Regarding major complications, 20 patients (7.2%) in the MOABP group and 40 patients (13.9%) in the MBP plus placebo group had Clavien-Dindo grade III or higher complications (*P* = .01). Similarly, patients in the MOABP group compared with the MBP plus placebo group experienced fewer SSIs (23 [8.3%] vs 48 [16.7%]; OR, 0.45 [95% CI, 0.27-0.77]) and fewer anastomotic dehiscences (16 [5.8%] vs 39 [13.5%]; OR, 0.39 [95% CI, 0.21-0.72]) (Table 4). The reduction in SSIs was mostly due to a decrease in organ and space SSIs, whereas the rates of superficial and deep incisional infections were similar between the 2 groups (Table 4). Differences in anastomotic dehiscence were most pronounced for grade A anastomotic dehiscence, which occurred in 5 patients (1.8%) in the MOABP group and 22 (7.6%) in the MBP group (Table 4). All but 1 patient who had grade A anastomotic dehiscence had undergone prophylactic proximal diversion in the primary operation. Two patients with grade A anastomotic dehiscence (1 in each group) had repeat surgery due to anastomotic dehiscence more than 30 days after the initial surgery. Length of hospital stay and rate of necessary adjuvant therapy were similar between the 2 groups (Table 4). Five patients died within 90 days after surgery; death was due to anastomotic dehiscence and peritonitis in 1 patient and myocardial infarction in 2 patients in the MOABP group, and due to aspiration pneumonia in 1 patient and cirrhosis of the liver with acute kidney injury in another patient in the MBP plus placebo group. Regarding the potential adverse effects of antibiotics, only 1 *Clostridioides difficile* infection was reported in the MOABP group and 1 patient in each group reported nausea after taking tablets. No allergic reactions were recorded.

**Table 4.** Primary and Secondary Outcomes of Patients in the MOABP and MBP Plus Placebo Groups

Outcome	No. (%)		Effect size		*P* value^a^
	MBP plus placebo (n = 288)	MOABP (n = 277)	OR (95% CI)	Wilcoxon effect size	
Comprehensive Complication Index^b^					
Mean (SD)	12.3 (15.3)	8.3 (13.9)			
Median (IQR)	8.66 (0-20.92)	0 (0-8.66)	NA	0.146	<.001
Surgical site infection	48 (16.7)	23 (8.3)	0.45 (0.27-0.77)	NA	.003
Superficial incisional	5 (1.7)	6 (2.2)	NA	NA	
Deep incisional	0	0	NA	NA	
Organ or space	43 (14.9)	17 (6.1)	NA	NA	
Anastomotic dehiscence^c^	39 (13.5)	16 (5.8)	0.39 (0.21-0.72)	NA	.002
Grade A	22 (7.6)	5 (1.8)	NA	NA	
Grade B	5 (1.7)	3 (1.1)	NA	NA	
Grade C	12 (4.2)	8 (2.9)	NA	NA	
Length of hospital stay, median (IQR), d^b^	6 (5-8)^d^	6 (5-7)^e^	NA	0.050	.23
Mortality within 90 d after surgery	2 (0.7)	3 (1.1)	1.57 (0.26-9.44)	NA	.68^f^
No. of patients receiving adjuvant treatment/total No. of patients requiring adjuvant treatment^g^	94/108 (87)	96/110 (87)	0.98 (0.44-2.17)	NA	.96

Abbreviations: MBP, mechanical bowel preparation; MOABP, mechanical and oral antibiotics bowel preparation; NA, not applicable; OR, odds ratio.

^a^ With Bonferroni correction, *P* < .01 is considered statistically significant for secondary outcomes.

^b^ Comprehensive Complications Index and length of hospital stay were not normally distributed, and hence effect size is given as Wilcoxon effect size (r = Z / [square root of N]) without 95% CIs and the *P* value is calculated using Mann-Whitney *U* test. Wilcoxon effect size of 0.1 is considered as a small effect, 0.3 as a moderate effect, and 0.5 and above as a large effect. The mean (SD) Comprehensive Complication Index is shown, but no statistical testing for mean difference was performed as it was not normally distributed.

^c^ Grade A: anastomotic dehiscence resulting in no change in patient’s management; grade B: dehiscence requiring active therapeutic intervention but no repeat laparotomy; grade C: anastomotic dehiscence requiring repeat laparotomy or laparoscopy.

^d^ Data for 1 patient were excluded (died during hospital stay).

^e^ Data for 2 patients were excluded (died during hospital stay).

^f^ Fisher exact test.

^g^ The criteria for adjuvant treatment were pathologic nodal positivity, lymphovascular invasion, high tumor budding, or high-grade adenocarcinoma.

In the prespecified subgroup analyses, MOABP reduced overall postoperative complications in patients with low rectal tumors, with no or only SCRT, with protective stoma, or undergoing open surgery (eTable 2 in Supplement 2). Reduction of overall postoperative complications in patients in the MOABP group was not demonstrated in the subgroups of patients with high rectal tumors, with LCCRT, without protective stoma, or in those undergoing minimally invasive surgery. Finally, MOABP reduced SSIs in patients with low rectal tumors or with no or only SCRT, but not in other subgroups (eTable 2 in Supplement 2).

## Discussion

In this double-blind, placebo-controlled randomized clinical trial involving patients undergoing elective rectal resection, MOABP resulted in fewer overall postoperative complications than MBP plus placebo (median CCI, 0 vs 8.66). The difference between the medians is comparable to 1 Clavien-Dindo grade I complication, which corresponds to a CCI score of 8.7. While this difference might seem minor, it is important to note that the rate of major complications was halved with MOABP (from 13.9% in the MBP plus placebo group to 7.2% in the MOABP group). Furthermore, the overall incidence of SSIs, and specifically anastomotic dehiscences (5.8% vs 13.5%) were reduced with use of MOABP. No major adverse events associated with oral antibiotics occurred, and only 1 *C. difficile* infection was noted. Subgroup analyses suggest that the largest benefit of MOABP may be seen in patients undergoing open low rectal resection.

To our knowledge, MOBILE2 is the first high-quality prospective trial comparing MOABP with MBP alone in patients undergoing elective rectal resection. However, 2 small, underpowered, unblinded RCTs with methodological concerns that included only patients undergoing rectal resection have been published.^26,27^ One of these studies was published 16 years after recruitment, and the other used inappropriate intravenous (cephalosporin only) and oral antibiotics (erythromycin and metronidazole). Both studies reported a reduction of SSIs in patients who received oral antibiotics. In addition, there are a few large trials comparing MOABP with MBP alone, but these trials did not report the outcomes of rectal or left-sided anastomoses separately. One trial included a large number of rectal anastomoses (181 patients) and reported a reduced SSI rate in the MOABP group, but there was no subgroup analysis with patients undergoing only rectal resection.^28^ Two studies with a recruitment period in the 1990s reported similar results, but did not report data separately for rectal resections.^29,30^ A recent trial in which selective digestive decontamination was used with MBP for left-sided bowel resections (including rectal resections in 23% of patients) reported fewer infectious complications, but no difference in anastomotic dehiscences, in the decontamination group.^31^ There are also a handful of small trials including different types and locations of colorectal anastomoses. Although left-sided anastomoses in these trials usually also include rectal resections, they also include left-sided colectomies and sigmoid resections, which have different complication risks. For example, the subgroup analysis of the MOBILE1 trial included only left-sided colectomies and did not find any difference in overall postoperative complications or SSIs in patients in the MOABP vs the no bowel preparation group.^32^ Accordingly, a recent review concluded that the applicability of these results is limited because most studies include patients undergoing both colon and rectal resections.^15^ It is likely that patients with right-sided colectomies, left-sided colectomies, and rectal resections benefit from different bowel preparation regimens.

### Limitations

This study has limitations. First, some groups in the subgroup analyses were relatively small; thus, the data may suffer from false-negative (ie, type II) error. Hence, the lack of demonstrated benefit in the subgroup analyses should not be interpreted as evidence of no benefit in these groups. Second, the use of minimally invasive approaches was relatively uncommon and the use of protective stoma relatively common. Differences in the rates of these methods may affect outcomes. The benefit of MOABP might not be as prominent in minimally invasive surgery as in open surgery or in upper rectal resections, which have a lower risk for SSIs and anastomotic dehiscence. Third, patients in both groups received MBP, which might affect SSI rates. And finally, surgeons reporting SSIs in their own patients may be considered a limitation, even though in our double-blinded study, this limitation affects both groups equally.

## Conclusions

The results of this randomized clinical trial indicate that MOABP resulted in fewer overall postoperative complications as well as fewer SSIs and anastomotic dehiscences in patients undergoing elective rectal resection compared with MBP alone. Based on these findings, MOABP should be considered as standard treatment in patients undergoing elective rectal resection. Further follow-up of the patients included in this trial will shed light on the long-term, especially oncological, outcomes after MOABP vs MBP.

## Notes

Supplement 1.
Trial Protocol and Statistical Analysis

Supplement 2. **eTable 1.** List of Specific Complications Within 30 Days After Surgery **eTable 2.** Prespecified Subgroup Analyses for Overall Postoperative Complications (CCI) and SSIs **eTable 3.** Neoadjuvant Treatment According to Tumor Stage

Supplement 3.
Data Sharing Statement
