# Supplementary material for: Morbidity After Mechanical Bowel Preparation and Oral Antibiotics Prior to Rectal Resection: The MOBILE2 Randomized Clinical Trial
Source: JAMA Surg. 2024 Mar 20;159(6):606–14. doi: 10.1001/jamasurg.2024.0184 (PMC10955353; doi:10.1001/jamasurg.2024.0184)
Supplement: Supplement 1. — Trial Protocol and Statistical Analysis [file jamasurg-e240184-s001.pdf]

Research plan 17.03.2020, version 1.6 /Translated by Translation Agency Apropos Lingua

Mechanical Bowel Preparation and Oral Antibiotics Versus Mechanical Bowel Preparation Only Prior Rectal Surgery (MOBILE2): a prospective randomized study

Laura Koskenvuo, Pipsa Lunkka, Pirita Varpe, Marja Hyöty, Reetta Satokari, Carola Haapamäki, Anna Lepistö, Ville Sallinen

There is a long history of research into the benefits of preparing the bowel before colorectal cancer surgery. Traditionally, the bowel has been prepared mechanically by drinking a cleansing solution before the colorectal surgery (Nichols RL et al 1997). Cleansing of the bowel was thought to reduce surgical site infections and the risk of anastomotic leakage. However, it has since been concluded that cleansing of the bowel does not, in itself, reduce the quantity of bacteria in the intestinal mucosa. After the mechanical preparation of the bowel, it could be beneficial to provide the patient with oral antibiotics, which would reduce the number of bacteria in the intestinal mucosa; in practice, the cleansing of the bowel simply allows the antibiotic to reach the mucosa (Fry DE, et al, 2011). The mechanical preparation process causes discomfort to the patients, and, in particular, elderly patients may be at risk of electrolyte imbalance and dehydration. In recent years, extensive series of studies questioning the benefits of cleansing the bowel have been published (Ram E et al 2005). Therefore, the practice of cleansing the bowel before colon surgery has mainly been stopped. For rectal surgery, the situation is different. The risk of leakage is higher for low colorectal or coloanal anastomoses, and on the other hand, a protective stoma is often created for the patient. In these cases, the section of bowel between the stoma and the anastomosis has been cleansed by emptying the bowel before surgery. At one point, a subgroup analysis of a meta-analysis was published stating that mechanical bowel preparation offers no benefit in rectal surgery, either (Guenaga et al 2009). However, a French research group showed that mechanical bowel preparation does reduce infectious complications after rectal surgery (34% vs. 16%) (Bretagnol et al 2010). Currently, in large

cancer centres, the bowel is typically emptied before rectal surgery as a precautionary measure in the absence of a broader body of research.

Recently, several extensive retrospective series focusing on colorectal patients have been published where patients that underwent bowel cleansing and received oral (PO) antibiotics have been compared with patients that received no bowel preparation before surgery. These studies have shown a clear difference in favour of the patients that received bowel preparation measures (Morris MS et al. 2015, Kim EK et al. 2014, Scarborough JE 2015, Toneva GD et al. 2013). In addition, in a study focusing on left colon and rectal resection patients, the difference in favour of the bowel preparation group was even clearer (8% vs. 27%) (Vo et al, 2018).

According to an earlier meta-analysis, a combination of mechanical bowel preparation and oral antibiotics, as compared to mechanical preparation without antibiotics, reduces surgical site infections, but not total complications or leakages. (Rollins et al 2018) We are not aware of any randomized studies comparing these approaches while only including rectal resection patients. We are also not aware of any studies looking at the effects that this type of bowel preparation has on the bacterial composition of the colon.

The treatment of rectal cancers in Southern Finland has been centralized in the Helsinki University Hospital, Abdominal Centre, Department of Colorectal surgery. About 300 rectal cancer surgeries take place at the centre annually. Other research locations include the Turku University Hospital Digestive Surgery and Urology Department and the Tampere University Hospital Digestive Surgery Clinic. Other Finnish university and central hospitals may also join the study later on.

Hypothesis

Using per oral antibiotics for bowel preparation after mechanical bowel preparation reduces surgical site infections compared to traditional bowel preparation where per oral antibiotics are not used.

Methods

Locations

Helsinki University Hospital, Abdominal Centre, Department of Colorectal surgery

Turku University Hospital, Digestive Surgery and Urology Department

Tampere University Hospital, Digestive Surgery Clinic

Other Finnish university and central hospitals may join the study later

Intervention groups

1. Bowel preparation using mechanical bowel preparation and PO antibiotics
2. Bowel preparation using mechanical bowel preparation and placebo

Research patients

Inclusion criteria

1. Patients coming in for an anterior resection (resection of the rectum and performing colorectal or coloanal anastomosis) due to a rectal tumour.

Exclusion criteria

1. Emergency surgery
2. Intestinal obstruction before the surgery (mechanical bowel preparation not possible)
3. Stoma before the surgery (mechanical bowel preparation not possible)

4. Any other reason preventing mechanical bowel preparation
5. Allergy to the antibiotics used in the study
6. Age <18
7. Inadequate ability to co-operate
8. Patient is not willing to participate

Post-randomization exclusion criteria (patients will be excluded from the analyses if even one of these conditions is met)

1. The surgery was not performed
2. The rectal resection was not performed
3. A colon anastomosis was not created (for example, an end colostomy was performed instead).

Randomization

The patients are randomized 1:1 between the preparation group and the no preparation group. The randomization sequence will be generated with a computer, using variable block size. The patient population will be stratified according to the distance of the lower edge of the tumour from the anal verge (measured from rectal MRI) and, on the other hand, according to the preoperative treatment they receive. Four different groups will be created.

Group A: <10 cm from the anal verge, short radiotherapy with immediate surgery or no preoperative treatment
Group B: <10 cm from the anal verge, chemoradiotherapy or short radiotherapy + long waiting time
Group C: ≥10 cm from the anal verge, short radiotherapy with immediate surgery or no preoperative treatment
Group D: ≥10 cm from the anal verge, chemoradiotherapy or short radiotherapy + long waiting time

Randomization will take place at the ward, in connection with the preoperative appointment, after the patient has first given their written consent to participate in the study. Randomization is done using numbered closed envelopes.

Blinding

The study will be a double-blinded. Patients in both groups will undergo mechanical bowel preparation. Control-group patients will receive a placebo that looks identical to the medication given to the other group. Therefore, patients, treating physicians, ward nurses, data collectors and outcome analysers will be blinded for the allocated treatment. Once all the data has been collected and is in the analysis phase, patients will be sorted to groups A and B, but these will not yet be connected to whether the patient received a placebo or an antibiotic. Only after the results have been analysed for the main and secondary variables will the blinding be completely unmasked.

Patients will undergo mechanical bowel preparation and take their medication at the ward or at home, according to the nurses' instructions. "Emergency envelopes" will be stored in the doctor's office at the ward, at a specific marked location, in case information about which medicine the patient has received is suddenly needed during treatment.

Intervention

1. Bowel preparation using mechanical bowel preparation and PO antibiotics.

   The mechanical bowel preparation will be performed using Moviprep, which contains polyethylene glycol (PEG) and is routinely used in Helsinki University Hospital. The patient will drink 2 litres of Moviprep and 1 litre of clear fluids of the patient's choice. The mechanical bowel preparation can be started two days before the surgery at 15:00 and it must be completed by 15:00 on the day before the surgery. After this, the patient will take 1 g of neomycin and 1 g of metronidazole orally at 15:00 and 23:00. Patients will receive a perioperative prophylactic IV antibiotic 1 hour before surgery and, if surgery is ongoing at 3 hours after the first dose, the antibiotic dose is repeated (cefuroxime 1.5 g, metronidazole

500 mg).

2. Bowel preparation using mechanical bowel preparation and placebo.

   The mechanical bowel preparation will be performed using Moviprep, which contains polyethylene glycol (PEG) and is routinely used in Helsinki University Hospital. The patient will drink 2 litres of Moviprep and 1 litre of clear fluids of the patient's choice on the day before the operation. The mechanical bowel preparation process can be started two days before the surgery at 15:00 and it must be completed by 15:00 on the day before the surgery. After this, the patient will take placebo 1 and placebo 2 orally at 15:00 and 23:00. Patients will receive a perioperative prophylactic IV antibiotic 1 hour before surgery and, if surgery is ongoing at 3 hours after the first dose, the antibiotic dose is repeated (cefuroxime 1.5 g, metronidazole 500 mg).

Outcomes

Primary outcome

Comprehensive complication index within 30 days of the operation (Slankamenac K et al 2013)

Secondary outcomes

1. Surgical site infection within 30 days of the procedure (according to CDC criteria), including
   a. Superficial incisional infection
   b. Deep incisional infection
   c. Organ / space infection
2. The number and classification of anastomosis leakages within 30 days of procedure

3. Length of the hospital stay
4. Mortality within 90 days of the procedure (any cause)
5. The number of patients that received adjuvant treatment divided by the number of patients that needed it within 6 months of the procedure. (Stage III and IV cancers, as well as stage II, if the cancer is poorly differentiated, grows into an adjacent organ, insufficient number of lymph nodes removed, inadequate healthy tissue margin, tumour perforation (Am Cancer Society recommendation, www.cancer.org))

Tertiary outcomes (long-term follow-up)

1. 5-year overall survival
2. 5-year disease specific survival
3. 5-year recurrence free survival
4. Difference in quality of life questionnaire (SF-36, QLQ-C30, QLQ-CR29, LARS score) scores before the surgery vs. 1 year after the surgery

Exploratory outcomes

1. Side effects of antibiotic treatment within 30 days of the procedure
2. Intestinal microbiome (only patients of the Helsinki University Hospital)

Follow-up

Normal post-rectal-cancer follow-up will be scheduled. At 6-8 weeks after surgery, the patients will have an imaging with contrast medium per rectum or, alternatively, a sigmoidoscopy. At this time, it is checked whether the patient has experienced any surgical site infections, other complications, reoperations or died. With regard to long-term outcomes (tertiary outcomes), recurrences are checked from patient records, if necessary, by ordering patient records from other hospitals, and the patient can be contacted by letter or telephone if necessary. Patients will also be asked to complete quality of life questionnaires (SF-36, QLQ-C30, QLQ-CR29) before surgery and at 1 year after the surgery.

Adverse events from the medication will be reported to Finnish Medicines Agency (Fimea) within the normal recommended time limits.

Costs

The costs of antibiotics and placebos, stool sample handling and time spent by researchers will be covered by the Abdominal Centre's research funding or external grants applied for by the researchers. The patients will not require extra visits or examinations due to participating in the study. The cost of the medicines is estimated to be around 2,000 euros, the cost of handling stool samples 1,000 euros and the time spent by the researchers is estimated to cost 9,500 euros.

Parameters to be collected

See CRF1-6, SF-36, QLQ-C30, QLQ-CR29, LARS score.

Intestinal microbiome analysis

Samples will be taken from the faeces in the colon as well as the mucous membrane biopsies of all patients randomized at Helsinki University Hospital. Samples will be taken at the planning visit, at the surgery and 6 and 12 months after the surgery and used for microbiome analysis and mucosa gene expression. The samples will be stored directly in RNALater solution for analysis.

Analyses: The intestinal microbiome analyses will be performed in collaboration with Adjunct professor Reetta Satokari's (University of Helsinki) research group. The primary analysis method will be high-throughput sequencing of the 16S rRNA genes of the bacteria. Profiling of 16S rRNA genes reveals the composition of the microbiome. Based on this information, a more detailed analysis using so-called metagenomic sequencing, in which the genetic material of the microbes is extensively sequenced and their properties can be elucidated, can be considered. The analyses will

be performed using methods the research group uses routinely. Adjunct proseffor Satokari's research group has extensive experience on intestinal microbiome composition studies (Jalanka et al 2016, Cheng et al 2016, Nylund et al 2015), therapeutic use of intestinal bacteria (faecal transplants) (Satokari et al 2015, Jalanka et al 2017) and studying host–microbe interactions (Hiippala et al 2016, Reunanen et al 2015).

Sample size

In our own data, the number of leakages/abscesses related to low anterior resections performed in 2005–2011 was 12.8%, but minor wound complications were not reported (Räsänen et al. 2015). In our previous randomized study comparing mechanical bowel preparation combined with PO antibiotics to a group where mechanical bowel preparation was not used at all, patients undergoing colon resection had leakage / abscess counts of approximately 6–8% and a Comprehensive Complication Index (CCI) of 9–10, SD 13–16. Based on these, we estimate that the Comprehensive Complication Index is higher in rectal surgery than in colon surgery and the SD may also be higher. Sample size was calculated with the aim of showing a difference of 5 CCI points between the groups (hypothesis: 12.5 points in the non-antibiotic group, 17.5 points in the antibiotic group). The SD is estimated to be 18 in both groups. With a power of 90% and a margin of error of 5%, 574 patients need to be sampled (Willcoxon-Mann-Whitney test). 5% of patients are estimated to disappear during follow-up, resulting in a final sample size of 604 patients.

Statistical analysis plan

The primary outcome will be analysed using either the Mann-Whitney U-test or the t-test, bootstrapped or log-transformed if necessary. Secondary outcomes will be analysed using either the t-test or the Mann-Whitney U-test for continuous variables, depending on the distribution, and using Chi-square or Fisher's exact test for categorical variables. If necessary, log-transformation can be performed on non-normally distributed continuous variables, or a bootstrapped t-test can be used. A more detailed classification of surgical site infections will be reported (superficial, deep, organ / space). Tertiary outcomes will be analysed separately at a later date, when at least 5-year follow-up

is available for all patients. Kaplan-Meier and the log-rank test will be used for analysis.

When reporting results, the following subgroup analyses will be performed for all outcome variables:

1) Tumour location (lower edge < or ≥ 10 cm from anal verge, based on pre-op MRI)
2) Long-term chemoradiotherapy before the surgery (yes/no)
3) Protective stoma (yes/no)
   [Amendment 29th April 2021 based on peer reviewer's comment during review process in BMJ Open : Added subgroup analysis group 4) Surgical approach (minimally invasive / open surgery)]

Schedule

The research project is scheduled to start on March, 2020. It is estimated that it will take about 2-3 years to reach the number of patients according to the power calculation. The follow-up phase is estimated to continue until the end of 2027.

Registration

The study will be registered in the ClinicalTrials.gov before randomization begins.

Data protection

During the study, patient identification data will be collected in a study folder. The data collected in the study is stored and analysed without patient identification data. At the randomization stage, each study patient will receive a study number, which will be stored with the patient's identification information in the study folder. The study number will be attached to the collected data, and the patient can be identified using the information in the study folder, if necessary. The material to be collected will be stored in a locked room, and electronic data will be stored on Helsinki University Hospital computers, on password-protected drives. Data will be processed in accordance with the

General Data Protection Regulation (GDPR) and the basis for processing the data will be Article 6 (e) in conjunction with Article 9 (i).

References

Nichols RL, Smith JW, Garcia RY, Waterman RS, Holmes JW. Current practices of preoperative bowel preparation among North American colorectal surgeons. Clin Infect Dis. 24:609-19, 1997.

Fry DE. Colon preparation and surgical site infection. Am J Surg. 202:225-232. 2011.

Ram E, Sherman Y, Weil R, et al. Is mechanical bowel preparation mandatory

for elective colon surgery? Arch Surg. 140:285-288, 2005.

Guenaga KK, Matos D, Wille J, Orgensen P. Mechanical bowel preparation for elective colorectal surgery. Cochrane Database Syst Rev. 1:CD001544, 2009.

Bretagnol F, Panis Y, Rullier E et al. Rectal Cancer Surgery With or Without Bowel Preparation: The French Greccar III Multicenter Single-Blinded Randomized Trial. Annals of Surgery. 252:863-868, 2010.

Morris, MS, Graham, LA, Chu, DI, Cannon, JA, Hawn, MT. Oral Antibiotic Bowel Preparation Significantly Reduces Surgical Site Infection Rates and Readmission Rates in Elective Colorectal Surgery. Ann Surg. 261:1034-40, 2015.

Kim EK, Sheetz KH, Bonn J, et al. A statewide colectomy experience: the

role of full bowel preparation in preventing surgical site infection. Ann Surg. 259:310-314, 2014.

Scarborough JE, Mantyh CR, Sun Z, Migaly J. Combined mechanical and oral antibiotic bowel preparation reduces incisional surgical site infection and anastomotic leak rates after elective colorectal resection: An analysis of colectomy-targeted ACS NSQIP. Ann Surg. 262:331-7, 2015.

Toneva GD, Deierhoi RJ, Morris M, et al. Oral antibiotic bowel preparation

reduces length of stay and readmissions after colorectal surgery. J Am Coll Surg. 216:756-763, 2013.

JAMA Surg. 2018 Feb 1;153(2):114-121. doi: 10.1001/jamasurg.2017.3827.

Vo E, Massarweh NN, Chai CY, Tran Cao HS, Zamani N, Abraham S, Adigun K, Awad SS.

Association of the Addition of Oral Antibiotics to Mechanical Bowel Preparation for Left Colon and Rectal Cancer Resections With Reduction of Surgical Site Infections. JAMA Surg. 153:114-121, 2018.

Rollins KE, Javanmard-Emamghissi H, Acheson AG, Lobo DN. The Role of Oral Antibiotic Preparation in Elective Colorectal Surgery: A Meta-analysis. Ann Surg. 2019 Jul;270(1):43-58.

Slankamenac K, Graf R, Barkun J, Puhan MA, Clavien PA. The comprehensive complication index: a novel continuous scale to measure surgical morbidity. Ann Surg. 258:1-7, 2013.

Jalanka J, Mattila E, Jouhten H, Hartman J, de Vos WM, Arkkila P, Satokari R.

Long-term effects on luminal and mucosal microbiota and commonly acquired taxa in faecal microbiota transplantation for recurrent Clostridium difficile infection.

BMC Med. 14:155, 2016.

Cheng J, Ringel-Kulka T, Heikamp-de Jong I, Ringel Y, Carroll I, de Vos WM, Salojärvi J, Satokari R.

Discordant temporal development of bacterial phyla and the emergence of core in the fecal microbiota of young children. ISME J. 10:1002-14, 2016.

Nylund L, Nermes M, Isolauri E, Salminen S, de Vos WM, Satokari R.

Severity of atopic disease inversely correlates with intestinal microbiota diversity and butyrate-producing bacteria. Allergy. 70:241-4, 2015.

Satokari R, Mattila E, Kainulainen V, Arkkila PE. Simple faecal preparation and efficacy of frozen inoculum in faecal microbiota transplantation for recurrent Clostridium difficile infection--an observational cohort study. Aliment Pharmacol Ther. 41:46-53, 2015.

Jalanka J, Hillamaa A, Satokari R, Mattila E, Anttila VJ, Arkkila P. The long-term effects of faecal microbiota transplantation for gastrointestinal symptoms and general health in patients with recurrent Clostridium difficile infection. Aliment Pharmacol Ther. 47:371-379, 2018.

Lahtinen P, Mattila E, Anttila VJ, Tillonen J, Teittinen M, Nevalainen P, Salminen S, Satokari R, Arkkila P.

Faecal microbiota transplantation in patients with Clostridium difficile and significant comorbidities as well as in patients with new indications: A case series. World J Gastroenterol. 23:7174-7184, 2017.

Hiippala K, Kainulainen V, Kalliomäki M, Arkkila P, Satokari R. Mucosal Prevalence and Interactions with the Epithelium Indicate Commensalism of Sutterella spp. Front Microbiol. 7:1706. eCollection 2016.

Reunanen J, Kainulainen V, Huuskonen L, Ottman N, Belzer C, Huhtinen H, de Vos WM, Satokari R.

Akkermansia muciniphila Adheres to Enterocytes and Strengthens the Integrity of the Epithelial Cell Layer. Appl Environ Microbiol. 81:3655-62, 2015.

Räsänen M, Carpelan-Holmström M, Mustonen H, Renkonen-Sinisalo L, Lepistö A. Pattern of rectal cancer recurrence after curative surgery. Int J Colorectal Dis. 30:775-85, 2015.
