# Supplementary material for: Morbidity After Mechanical Bowel Preparation and Oral Antibiotics Prior to Rectal Resection: The MOBILE2 Randomized Clinical Trial
Source: JAMA Surg. 2024 Mar 20;159(6):606–14. doi: 10.1001/jamasurg.2024.0184 (PMC10955353; doi:10.1001/jamasurg.2024.0184)
Supplement: Supplement 2. — eTable 1. List of Specific Complications Within 30 Days After Surgery eTable 2. Prespecified Subgroup Analyses for Overall Postoperative Complications (CCI) and SSIs eTable 3. Neoadjuvant Treatment According to Tumor Stage [file jamasurg-e240184-s002.pdf]

Supplemental Online Content

Koskenvuo L, Lunkka P, Varpe P, et al. Morbidity after mechanical bowel preparation and oral antibiotics prior to rectal resection (MOBILE2): a randomized clinical trial. *JAMA Surg.* Published online March 20, 2024. doi:10.1001/jamasurg.2024.0184

eTable 1. List of Specific Complications Within 30 Days After Surgery

eTable 2. Prespecified Subgroup Analyses for Overall Postoperative Complications (CCI) and SSIs

eTable 3. Neoadjuvant Treatment According to Tumor Stage

This supplemental material has been provided by the authors to give readers additional information about their work.

eTable 1. List of Specific Complications Within 30 Days After Surgery

Clavien-Dindo grade and type - no. (%)	Mechanical bowel preparation and placebo (N=288)	Mechanical and oral antibiotic bowel preparation (N=277)
None	***134 (46.5%)***	***155 (56.0%)***
1 (one or more per patient)	***116 (40.3%)***	***109 (39.4%)***
Wound infection	5 (1.7)	2 (0.7%)
Anastomotic dehiscence	1 (0.3%)	0
Ileus (vomiting, nasogastric tube placement)	51 (17.7%)	48 (17.3%)
Electrolyte imbalance	16 (5.6%)	17 (6.1%)
Collapse	4 (1.4%)	5 (1.8%)
Urinary retention	13 (4.5%)	10 (3.6%)
Incisional site bleeding (wound or anastomosis)	4 (1.4%)	5 (1.8%)
Incision resuturation	2 (0.7%)	1 (0.4%)
Abnormal intensive incisional or operation site pain	5 (1.7%)	1 (0.4%)
Fever	5 (1.7%)	4 (1.4%)
Problem with stoma site	3 (1.0%)	8 (2.9%)
Delirium	1 (0.3%)	1 (0.4%)
Shortness of breath, Pain in chest	6 (2.1%)	5 (1.8%)
Peroneal nerve Paralysis	0	1 (0.4%)
2 (one or more per patient)	***81 (28.1%)***	***47 (17.0%)***
Anastomotic dehiscence	1 (0.3%)	0
Abscess at anastomotic site	2 (0.7%)	1 (0.4%)
Wound infection	3 (1.0%)	4 (1.4%)
Prolonged ileus with medication	11 (3.8%)	6 (2.2%)
Clostridium difficile infection	0	1 (0.4%)
Incisional site bleeding (wound or anastomosis)	13 (4.5%)	4 (1.4%)
Urinary tract infection	14 (4.9%)	6 (2.2%)
Pneumonia	6 (2.1%)	5 (1.8%)
Urinary retention	4 (1.4%)	4 (1.4%)
Pulmonary embolism	6 (2.1%)	3 (1.1%)
TIA	2 (0.7%)	0
Shortness of breath, Pain in chest	3 (1.0%)	2 (0.7%)
Other infection (bacteremia, cholecystitis)	4 (1.4%)	2 (0.7%)

Atrial fibrillation	2 (0.7%)	3 (1.1%)
Delirium	2 (0.7%)	0
Incisional site pain	1 (0.3%)	0
Collapse	0	1 (0.4%)
Fever	7 (2.4%)	6 (2.2%)
3a (one or more per patient)	***16 (5.6%)***	***8 (2.9%)***
Anastomotic dehiscence, endoscopic management<br>Abscess at anastomotic site, drainage	9 (3.1%)<br><br>3 (1.0%)	2 (0.7%)<br><br>2 (0.7%)
Prolonged ileus with intervention	1 (0.3%)	1 (0.4%)
Incisional site bleeding (wound or anastomosis)	1 (0.3%)	3 (1.1%)
Ascites<br>Headache, epidural blood patch	1 (0.3%)<br>1 (0.3%)	0<br>0
3b (one or more per patient)	***23 (8.0%)***	***10 (3.6%)***
Anastomotic dehiscence<br>Abscess at anastomotic site<br>Colonic perforation above anastomosis	9 (3.1%)<br>2 (0.7%)<br>1 (0.3%)	6 (2.2%)
Fascial dehiscence	3 (1.0%)	2 (0.7%)
Stoma revision	3 (1.0%)	1 (0.4%)
Prolonged ileus with intervention	2 (0.7%)	0
Incisional site bleeding (wound or anastomosis)<br>Intestinal strangulation	1 (0.3%)<br><br>1 (0.3%)	0
Intestinal perforation	1 (0.3%)	0
Incision resuturation	0	1 (0.4%)
4a (one or more per patient)	***7 (3.1%)***	***5 (1.8%)***
Anastomotic dehiscence<br>Dyspnea<br>Epileptic seizure<br>Pneumonia<br>AKI<br>Hypotension<br>Cardiac failure<br>Intra-abdominal bleeding	1 (0.3%)<br>3 (1.0%)<br>1 (0.3%)<br>1 (0.3%)<br>1 (0.3%)<br>0<br>0<br>0	1 (0.4%)<br>1 (0.4%)<br>0<br>0<br>0<br>1 (0.4%)<br>1 (0.4%)<br>1 (0.4%)
4b (one or more per patient)	***4 (1.4%)***	***0***

Anastomotic dehiscence	4 (1.4%)	0
5 (equals death)	***1 (0.3%)***[a]	***2 (0.7%)***[a]
Acute myocardial infarction	0	1 (0.4%)
Anastomotic dehiscence	0	1 (0.4%)
Aspiration pneumonia	1 (0.3%)	0

Data is presented as numbers (%s).

TIA, transient ischemic attack; AKI, acute kidney injury.

[a]A total of five patients died within 90 days, but this table shows complications and mortality up to 30 days.

eTable 2. Prespecified Subgroup Analyses for Overall Postoperative Complications (CCI) and SSIs

Subgroup		Mechanical bowel preparation and placebo	Mechanical and oral antibiotic bowel preparation	Effect size (OR [95% CI] or r)	p value[b]
Tumor location					
**Lower edge < 10cm from the anal verge**		N=192	N=191		
	CCI[a], median (IQR)	8.6603 (0-22.21)	0 (0-8.66)	0.8147	**0.0003**
	SSI, n (%)	40 (20.8)	15 (7.9)	0.324 (0.172-0.609)	**0.0003**
	Superficial	3 (1.6%)	5 (2.6%)		
	Deep	3 (1.6%)	1 (0.5%)		
	Organ	34 (17.7%)	9 (4.7%)		
**Lower edge ≥ 10cm from the anal verge**		N=96	N=86		
	CCI[a], median (IQR)	0 (0-20.92)	0 (0-9.56)	0.0749	0.312
	SSI, n (%)	8 (8.3)	8 (9.3)	1.128 (0.404-3.148)	0.818
	Superficial	2 (2.1%)	1 (1.2%)		
	Deep	1 (1.0%)	0		
	Organ/space	5 (5.2%)	7 (8.1%)		
Neoadjuvant treatment					
**No or short-course radiotherapy with immediate surgery**		N=233	N=224		
	CCI[a], median (IQR)	8.6603 (0-20.92)	0 (0-8.66)	0.1725	**0.0003**
	SSI, n (%)	40 (17.2)	12 (5.4)	0.273 (0.139-0.536)	**<0.0001**
	Superficial	5 (2.1%)	3 (1.3%)		
	Deep	3 (1.3%)	0		
	Organ/space	32 (13.7%)	9 (4.0%)		
**Yes**		N=55	N=53		
	CCI[a], median (IQR)	0 (0-20.92)	0 (0-14.79)	0.0299	0.758
	SSI, n (%)	8 (14.5)	11 (20.8)	1.539 (0.565-4.188)	0.397
	Superficial	0	3 (5.7%)		
	Deep	1 (1.8%)	1 (1.9%)		

	Organ/space	7 (12.7%)	7 (13.2%)		
Protective ostomy					
**Yes**		N=222	N=196		
	CCI[a], median (IQR)	8.6603 (0-22.64)	0 (0-8.66)	0.1865	**0.0001**
	SSI, n (%)<br>Superficial<br>Deep<br>Organ/space	40 (18.0)<br>3 (1.4%)<br>4 (1.8%)<br>33 (14.9%)	16 (8.2)<br>5 (2.6%)<br>1 (0.5%)<br>10 (5.1%)	0.404 (0.219-0.748)	0.0032
**No**		N=66	N=81		
	CCI[a], median (IQR)	0 (0-20.92)	0 (0-12.25)	0.0065	0.937
	SSI, n (%)<br>Superficial<br>Deep<br>Organ/space	8 (12.1)<br>2 (3.0%)<br>0<br>6 (9.1%)	7 (8.6)<br>1 (1.2%)<br>0<br>6 (7.3%)	0.686 (0.235-2.001)	0.488
**Surgical approach**					
**Open**		N=226	N=214		
	CCI[a], median (IQR)	8.6603 (0-21.35)	0 (0-9.56)	0.1498	**0.002**
	SSI, n (%)<br>Superficial<br>Deep<br>Organ/space	40 (17.7)<br>4 (1.8%)<br>3 (1.3%)<br>33 (14.6%)	18 (8.4)<br>6 (2.8%)<br>1 (0.5%)<br>11 (5.1%)	0.427 (0.236-0.771)	0.0040
**Minimally invasive**		N=62	N=63		
	CCI[a], median (IQR)	0 (0-20.92)	0 (0-8.66)	0.1325	0.139
	SSI, n (%)<br>Superficial<br>Deep<br>Organ/space	8 (12.9)<br>1 (1.6%)<br>1 (1.6%)<br>6 (9.7%)	5 (7.9)<br>0<br>0<br>5 (7.9%)	0.582 (0.179-1.888)	0.363

Data is presented as medians with IQRs or numbers (%s).

CCI, comprehensive complication index; SSI, surgical site infection; OR, odds ratio; CI, confidence interval; IQR, interquartile range.

[a]Not normally distributed; hence, effect size is given as Wilcoxon effect size

[b]After Bonferroni correction for multiple comparisons, a p-value < 0.003125 is considered statistically significant (bold).

eTable 3. Neoadjuvant Treatment According to Tumor Stage

	Stage 1 n (%)	Stage 2 n (%)	Stage 3 n (%)	Stage 4 n (%)	All n (%)
No	105 (99.1%)	88 (84.6%)	125 (45.6%)	9 (36.0%)	327 (64.2%)
Short cource radiotherapy	0	7 (6.7%)	59 (21.5%)	5 (20.0%)	71 (13.9%)
Short cource radiotherapy with delay	0	4 (3.8%)	3 (1.1%)	0	7 (1.4%)
Chemoradiotherapy	1 (0.9%)	5 (4.8%)	84 (30.7%)	6 (24.0%)	96 (18.9%)
Chemotherapy only	0	0	0	5 (20.0%)	5 (1.0%)
Total neoadjuvant treatment with short cource radiotherapy and chemotherapy	0	0	3 (1.1%)	0	3 (0.6%)
Total	106	104	274	25	509
