# Supplementary material for: Morbidity After Mechanical Bowel Preparation and Oral Antibiotics Prior to Rectal Resection: The MOBILE2 Randomized Clinical Trial
Source: JAMA Surg. 2024 Mar 20;159(6):606–14. doi: 10.1001/jamasurg.2024.0184 (PMC10955353; doi:10.1001/jamasurg.2024.0184)
Supplement: Supplement 3. — Data Sharing Statement [file jamasurg-e240184-s003.pdf]

Data Sharing Statement

Koskenvuo. Morbidity After Mechanical Bowel Preparation and Oral Antibiotics Prior to Rectal Resection (MOBILE2). *JAMA Surg*. Published March 20, 2024.
doi:10.1001/jamasurg.2024.0184

Data
Data available: No

Additional Information
Explanation for why data not available: The data may be shared in de-identified form if appropriate permits are sought and obtained.
